# Post cardiac surgery stunning reduces stroke work, but leaves cardiac power output unchanged in patients with normal ejection fraction

**DOI:** 10.14814/phy2.13781

**Published:** 2018-07-12

**Authors:** Tomas D. Tannvik, Audun E. Rimehaug, Nils K. Skjærvold, Idar Kirkeby‐Garstad

**Affiliations:** ^1^ Department of Anaesthesia and Intensive Care St Olav's Hospital Trondheim University Hospital Trondheim Norway; ^2^ Faculty of Medicine and Health Sciences Institute of Circulation and Medical Imaging Norges Teknisk‐Naturvitenskapelige Universitet Trondheim Norway

**Keywords:** Cardiac power, cardiac power output, cardiac stunning, stroke work

## Abstract

This study assesses positional changes in cardiac power output and stroke work compared with classic hemodynamic variables, measured before and after elective coronary artery bypass graft surgery. The hypothesis was that cardiac power output was altered in relation to cardiac stunning. The study is a retrospective analysis of data from two previous studies performed in a tertiary care university hospital. Thirty‐six patients scheduled for elective coronary artery bypass graft surgery, with relatively preserved left ventricular function, were included. A pulmonary artery catheter and a radial artery catheter were placed preoperatively. Cardiac power output and stroke work were calculated through thermodilution both supine and standing prior to induction of anesthesia and again day one postoperatively. Virtually all systemic hemodynamic parameters changed significantly from pre‐ to postoperatively, and from supine to standing. Cardiac power output was maintained at 0.9–1.0 (±0.3) W both pre‐ and postoperatively and from supine to standing on both days. Stroke work fell from pre‐ to postoperatively from 1.1 to 0.8 J (*P* < 0.001), there was a significant fall in stroke work with positional change preoperatively from 1.1 to 0.9 J (*P* < 0.001). Postoperatively the stroke work remained at 0.8 J despite positional change. Cardiac power output was the only systemic hemodynamic variable which remained unaltered during all changes. Stroke work appears to be a more sensitive marker for temporary cardiovascular dysfunction than cardiac power output. Further studies should explore the relationship between stroke work and cardiac performance and whether cardiac power output is an autoregulated intrinsic physiological parameter.

## Introduction

The heart essentially drives the human circulation by delivering hydraulic energy into the aorta in the form of a pressurized volume. The amount of energy delivered relies upon the heart's ability to produce pressure and flow, ventriculoarterial coupling and the properties of the cardiovascular tree. Energy delivery may be quantified either as hydraulic energy per second, here named cardiac power and measured in Watts (W) (Cotter et al. 2003), or considered through stroke work (SW), the energy delivered per heartbeat measured in Joules (J). Cardiac power output (CPO) is an estimate of cardiac power, calculated as the product of mean cardiac output (CO) and mean arterial pressure (MAP) (Westerhof et al. 2010). A mathematical approximation of stroke work is the product of mean stroke volume (SV) and MAP.

Although the clinical use of cardiac power variables is not common, they have been used for prognostication and as a guide to treatment of critically ill patients. CPO has been shown to strongly predict mortality for patients with acute coronary syndrome and for patients in chronic heart failure (Fincke et al. 2004; Yildiz et al. 2017). For patients admitted to an intensive care unit (ICU) with cardiogenic shock, a substantial drop in cardiac power index (CPI = CPO/body surface area) was associated with an increased 28 day mortality (Torgersen et al. 2009). Similarly, survival was significantly associated with a higher stroke work index (stroke work/body surface area) in hemodynamically compromised patients admitted to ICU after major trauma (Martin et al. 2006). Cardiac power variables have also been used as overall hemodynamic guides in trauma resuscitation with associated survival benefit (Chang et al. 2000).

Logically, the total amount of energy available for circulating the body has great impact on outcome in severe circulatory failure. However, the role of power variables in the monitoring and treatment of patients with mild to moderate circulatory dysfunction have been less explored. As a first step, we investigated whether cardiac power variables could be used to identify mild to moderate circulatory dysfunction.

Cardiac stunning is a common phenomenon of temporary cardiac dysfunction, seen frequently in the early postoperative stages of cardiac surgery (Kloner et al. 2001). Associated with this temporary cardiovascular dysfunction is vasodilatation with decreased systemic vascular resistance (SVR) and decreased MAP (Carrel et al. 2000). This patient group has a complex cardiovascular dysfunction and is thus particularly well suited for inclusion into a study assessing the role of cardiac power variables in mild to moderate temporary circulatory dysfunction.

The main aim of this study is to assess the change in CPO and SW in relation to classic hemodynamic markers in patients with mild to moderate cardiovascular dysfunction.

## Methods

This study utilizes data from 36 patients who were recruited for two previous studies. The data were recorded with the patients’ informed consent in the time period 1996–2004. As data were gathered from two previous studies, no formal calculation of sample size was performed. Thirty‐four male and two female patients with normal left ventricular function prior to elective coronary artery bypass graft (CABG) surgery were originally recruited from St. Olav University Hospital, Trondheim. All patients had a left ventricular ejection fraction of at least 0.5 as determined by angiography. Exclusion criteria for both studies included acute operation, serious organ dysfunction and heart failure class 4 as per New York Heart Association. Table 1 shows the general patient characteristics.

**Table 1:** Patient characteristics

Age (year, mean ± SD)	62.4 ± 9.5
Male, *n* (%)	34 (94)
Ejection fraction (%, mean ± SD)	71 ± 8
Preoperative angina, *n* (%)	35 (97)
Preoperative unstable angina, *n* (%)	8 (22)
Single vessel coronary artery disease, *n* (%)	5 (13.9)
Double vessel coronary artery disease, *n* (%)	5 (13.9)
Triple vessel coronary artery disease, *n* (%)	26 (72.2)
Preoperative myocardial infarction, *n* (%)	12 (33.3)
Hypertension, *n* (%)	14 (39)
Diabetes, *n* (%)	3 (8)
Cerebrovascular disease, *n* (%)	1 (3)

*n*, number of patients.

The original two studies concerned various aspects of early patient mobilization post cardiac surgery and were published in 2005 and 2006 (Kirkeby‐Garstad et al. 2005, 2006). A separate application for alternative analysis of these de‐identified hemodynamic data was submitted to the regional ethics committee, who had no objection in regard to the reuse of old measurements for this purpose (REK‐midt 2015‐849).

Preoperatively, prior to induction of anesthesia, all patients had a pulmonary artery catheter and a peripheral radial artery cannula placed, standard perioperative monitoring such as 5‐lead ECG and pulse oximetry was also initiated. The perioperative variables are shown in Table 2.

**Table 2:** Perioperative variables

Pre and postoperative dopamine requirements, *n* (%)	1 (2.8)
Pre and postoperative noradrenaline requirements, *n* (%)	2 (5.6)
Duration of cardiopulmonary bypass, mins, mean (SD)	54.9 (21.4)
Duration of postoperative intubation, mins, mean (SD)	192.4 (69.4)
Positive fluid balance postop, liters, mean (SD)	3.7 (1.1)
Preoperative use of betablocker, *n* (%)	32 (88.9)
Use of postoperative Nitroprusside, *n* (%)	4 (11.1)
Use of postoperative morphine mg/kg administered pre measurement day 1 postop, mean (SD)	0.26 (0.11)
Perioperative myocardial infarction^a^, *n* (%)	0 (0.0)
30 day mortality, *n* (%)	0 (0.0)

SD, standard deviation; *n*, number of patients.

a Myocardial infarction defined in this study as ECG changes and serial measurements of ASAT with an ASAT/ALAT ratio above 2.

Cardiac power output and SW were calculated from data taken at rest and standing prior to induction of anesthesia and at day one postoperatively, whilst awake. The patients underwent their scheduled procedure as per hospital protocol, including the use of a heart‐lung machine and cardioplegia. Cardiac output was determined through thermodilution technique via the pulmonary artery catheter. Each measurement was repeated at least three times using normal saline, of approximately 20°C. The mean of these three measurements were used in the subsequent calculations. CPO and SW were calculated using the following formulae:

CPO=mean arterial pressure (mm Hg)×cardiac output (L/min)/451

SW=stroke volume (mL)×mean arterial pressure (mm Hg)/7.5

For statistical analysis, we used repeated measures ANOVA. We organized the data in a 2 × 2 matrix with day and clinical situation as factors. This enabled us to investigate the effects of day independent of position as well as position independent of day. We could also assess the interaction between these two factors. The statistical analysis was performed using the software SPSS ver 20 (SPSS Inc, Chicago, IL). The figures were produced using the software R (R Core Team (2015). R: A language and environment for statistical computing. R Foundation for Statistica Computing, Vienna, Austria. URL https://www.R-project.org/).

## Results

**Figure 1:**
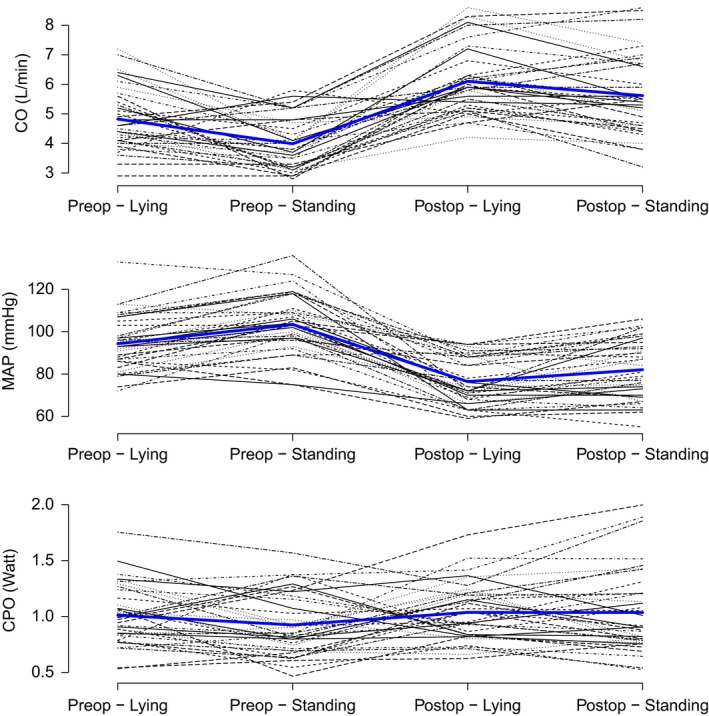
Cardiac output, mean arterial pressure and cardiac power output. Each variable shown pre and postoperatively for both lying and standing.

Paired comparisons of pre‐ and postoperative data, considering day showed that the CO increases, MAP decreases and CPO remains unchanged for both supine and standing (Tables 3 & 4 & Fig. 1). Paired comparisons of pre‐ and postoperative data, considering position, showed that the CO fell, MAP increased and CPO remains unchanged (Tables 3 & 4 & Fig. 1).

**Table 3:** Directly measured perioperative hemodynamic variables in mean (SD)

	Prior to induction of anesthesia		24 h postoperation		*P*‐value		
	Supine	Standing	Supine	Standing	Day	Sit	Inter
Cardiac output (L/min)	4.8 (1.0)	4.0 (0.9)	6.1 (1.2)	5.6 (1.4)	<0.001	<0.001	0.07
Mean arterial pressure (mm Hg)	94.3 (12.9)	103.4 (13.9)	76.4 (11.1)	82.0 (14.0)	<0.001	<0.001	0.11
Heart rate (per min)	56.6 (8.4)	63.6 (11.8)	75.1 (10.0)	81.5 (9.9)	<0.001	<0.001	0.56
CVP (mm Hg)	3.1 (2.0)	1.6 (2.4)	8.7 (4.2)	7.8 (4.8)	<0.001	0.002	0.42
PCWP (mm Hg)	9.3 (3.7)	5.8 (3.0)	12.6 (4.2)	11.8 (4.6)	<0.001	<0.001	0.02

SD, standard deviation; CVP, central venous pressure; PCWP, pulmonary capillary wedge pressure; mPAP, mean pulmonary arterial pressure; Sit, situation in terms of positional change; inter, interaction, in terms of assessing whether the magnitude of change in situation is different between days.

**Table 4:** Derived perioperative hemodynamic variables in mean (SD)

	Prior to induction of anesthesia		24 h postoperation		*P*‐value		
	Supine	Standing	Supine	Standing	Day	Sit	Inter
Cardiac power output (W)	1.0 (0.3)	0.9 (0.3)	1.0 (0.3)	1.0 (0.4)	0.14	0.18	0.10
Stroke work (J)	1.1 (0.3)	0.9 (0.2)	0.8 (0.2)	0.8 (0.2)	<0.001	<0.001	<0.001
Stroke volume (mL)	85.7 (16.8)	64.1 (15.4)	81.7 (13.3)	74.7 (12.2)	0.16	<0.001	<0.001
Systemic vascular resistance (dyne·sec/cm^5^)	1573.3 (367.5)	2113.5 (431.9)	911.0 (188.4)	1109.0 (317.5)	<0.001	<0.001	<0.001

SD, standard deviation; Sit, situation in terms of positional change; inter, interaction, in terms of assessing whether the magnitude of change in situation is different between days.

**Figure 2:**
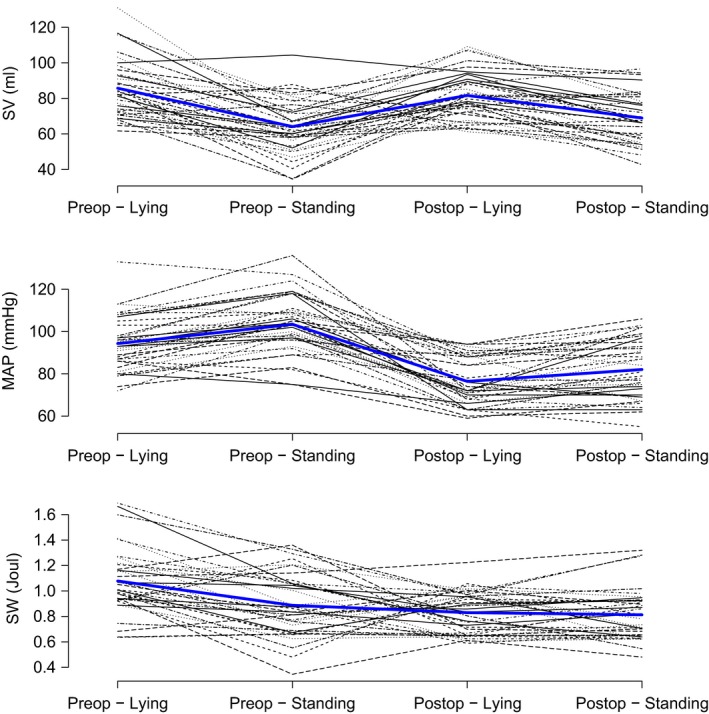
Stroke volume, mean arterial pressure, and stroke work. Each variable shown pre and postoperatively for both lying and standing.

In both the aforementioned situations, there is an increase in heart rate and a decrease in SW. The increase in heart rate is solely responsible for the increase in CO, as stroke volume is statistically unchanged from preoperatively to postoperatively. Despite an increase in MAP and a nonsignificant fall in SV, the net sum is a fall in SW (Fig. 2). There is a significant interaction between day and positional change for SW, SV, and SVR. Post hoc analysis, using paired *t*‐test showed a fall in SW with positional change preoperatively (*P* < 0.001) but not with positional change postoperatively (*P* = 0.607).

There is no significant difference in CPO pre and post‐CABG surgery (Table 4). Stroke work, however, did significantly vary from preoperative to postoperative.

## Discussion

The main finding of this study is that whilst MAP decreases CO appears to increase and together they yield a virtually unchanged CPO from preoperatively to day one postoperatively, and from supine to standing on both days. SW is significantly decreased, whilst CPO is maintained through an increase in heart rate in the early postoperative period. The relative stability of CPO appears to occur despite changes in all other clinically relevant systemic hemodynamic variables, except SV.

The rise in cardiac output between the 2 days and with positional change is due to the increase in heart rate. The fall in MAP from pre‐ to postoperatively must be seen in conjunction with the relatively high calculated fall in SVR. During cardiac surgery there will be some blood loss, which is partially replaced by crystalloids. It is postulated that this leads to a hemodilution which decreases the viscosity of the blood accounting for some of the decrease in SVR. Postoperative vasodilatation is also contributing to the decreased SVR (Carrel et al. 2000). The positive fluid balance, increased central venous pressure (CVP), increased pulmonary capillary wedge pressure (PCWP) and fall in SVR postoperatively may arguably be interpreted as signs of increased preload and decreased afterload. These responses would intuitively be predictors of an increased SV in the physiologically normal heart. This was not the case in the current study, where there was no difference in SV between the 2 days (*P* = 0.16). This indicates decreased cardiac performance, either due to systolic dysfunction and/or diastolic dysfunction. When calculating the SW, a feasible explanation to its decrease is due to cardiac stunning. Changes may also be due to alterations in ventriculoarterial coupling. The exact reason for this decrease in energy delivery per cardiac contraction cannot be explained using our data.

Despite postoperative cardiovascular dysfunction, no change in CPO was seen in our study population. Several factors may be postulated to be involved. All of the patients included in this study had a normal left ventricular systolic function preoperatively, as evaluated by left ventricular ejection fraction. In severe heart failure the baseline resting CPO is reduced (Yildiz et al. 2017). The amount of power that can be generated above the resting baseline is known as the cardiac reserve (Cotter et al. 2003; Klasnja et al. 2013). Based upon the preoperative state of the patients one has to consider whether the unchanged CPO is because the included patients had adequate cardiac reserve. It seems likely that CPO will significantly change in a patient population with a more pronounced cardiac failure preoperatively and hence less cardiac reserve. In a study by Tritapepe et al. on the effect of levosimendan on cardiovascular function after coronary artery bypass surgery the control group demonstrated subnormal cardiac power values on the first postoperative morning (Tritapepe et al. 2009). These patients had a lower preoperative EF than ours; also, they were not treated according to a fast‐track protocol.

On the other hand, SW appears more sensitive than CPO to show mild to moderately decreased cardiovascular function. The extent of cardiovascular impairment may be quantified using SW and monitored if necessary. As mentioned, cardiac power variables have been successfully used as a guide to treatment in trauma patients (Chang et al. 2000). Similarly, a stroke work index and CPO responsivenss to fluids were associated with survival in the general ICU population (Timmins et al. 1992). In the healthy population, a reference range for peak CPO using a noninvasive CO_2_ rebreathing technique has been calculated (Bromley et al. 2006). However, there is no common clinically verified normal reference range for SW calculated this way in a population undergoing coronary bypass graft surgery. Therefore, in this patient group, cardiac power variables should be performed preoperatively and multiple times postoperatively to observe trends in order to use these for clinical decision making, at least until a verified reference range is established. In term of treatment decisions, suggestions have been made that cardiac mechanical support should be initiated prior to cardiac power index falling below 0.34 W/m^2^ (Hall et al. 2012)_,_ CPI is considered normal from 0.5 to 0.7 (Vincent 2009).

Cardiac work has long been an interest for the cardiological community, using echocardiography based techniques (Russell et al. 2012; Vecera et al. 2016). The cardiological focus has traditionally been on the energy production and consumption within the heart whilst our group is predominantly interested in how much energy the heart can deliver to the body.

The general use of the pulmonary artery catheter has been declining internationally (Wiener and Welch 2007). The retrospective nature of this study was chosen for practical and ethical reasons. At Trondheim University Hospital, the pulmonary artery catheter is currently reserved for high‐risk patients with severely depressed myocardial function or requiring valve surgery, hence not our target population for this study. The authors also did not find it ethically sound to repeat invasive measurements in new patients with normal left ventricular systolic function when these data already existed from our institution. The included patients were collected retrospectively from two separate previous trials. This may have lead to unintended selection bias. In one of the two studies, only males were included in order to reduce heterogeneity. In the other study, few females were recruited due to which the patient population for elective CABG were mostly males. This may have lead to bias, however, we do not expect these hemodynamic variables to be highly dependent on sex.

For obvious clinical and ethical reasons, we could not induce actual hypovolemia to observe how power parameters fare under such conditions. Hence, moving from the supine position to standing is a low risk simulation of decreased preload due to hypovolemia. This has been shown in healthy individuals to significantly decrease cardiac output and mixed venous oxygen saturations (Harms et al. 2003). Another shortcoming of the study is that the injectate used for thermodilution was at room temperature, rather than cold. This could lead to decreased precision in regard to the calculations. However, it is important to note is that the measurements were repeated and averaged to increase precision. The use of tepid injectate was shown in one study to give a precision of cardiac output of 12.5% if the patient is spontaneously breathing and four measurements are performed (Kirkeby‐Garstad et al. 2015).

## Conclusion

Based on this study, we may hypothesize that patients with sufficient cardiac reserve increase heart rate to maintain CPO, making SW more suitable than CPO to track individual clinical development and response to treatment in mild to moderate cardiovascular dysfunction. One may postulate that as long as the heart can increase its heart rate to compensate for decreasing SW, the CPO will not fall. In severe heart failure, the SW may fall to a level in which the heart rate can no longer compensate through tachycardia and then a fall in CPO will be seen, reflecting the poor prognosis of patients with low cardiac power as seen in previous studies (Fincke et al. 2004; Torgersen et al. 2009). Future studies should further investigate how the decrease in SW relates to the degree of cardiovascular impairment and to clinical outcomes. More work is also required to investigate whether there exists an intrinsic autoregulation of CPO. We suggest considering cardiac power variables as performance based measurements to evaluate progress in the postoperative cardiothoracic ICU.

## Conflict of Interest

None declared.
